# A Case of the Large Cell Neuroendocrine Carcinoma of the Urinary Bladder

**DOI:** 10.1155/2013/804136

**Published:** 2013-04-04

**Authors:** Shinro Hata, Yoshihisa Tasaki

**Affiliations:** Department of Urology, National Hospital Organization Beppu Medical Center, 1473 Uchikamado, Beppu City, Oita 874-0011, Japan

## Abstract

Large cell neuroendocrine carcinoma (LCNEC) of the urinary bladder is very rare. Definite treatment strategy has not been established and prognosis of the disease is not clear yet. We report a case of primary LCNEC of the urinary bladder here with some review of the literature. The patient was a 84-year-old man. He underwent transurethral resection of bladder tumor (TURBT). Histological examination revealed a rosette arrangement of the tumor cells by HE staining and immunohistochemical study revealed positive CD 56, synaptophysin, and chromogranin A (LCNEC). After TURBT, he has no sign of recurrence for 8 months. We have to strictly observe the progress because LCNEC is very aggressive.

## 1. Introduction

Neuroendocrine carcinoma is extremely rare, accounting for less than 1% of bladder malignancy cases, with those developing in the bladder mainly being small cell carcinomas. In recent years, large cell neuroendocrine carcinomas (LCNEC) that differ in cell morphology compared with small cell carcinoma have been occasionally reported [1]. A case of LCNEC of the urinary bladder is presented in this case report.

## 2. Case Presentation

**Figure 1:**
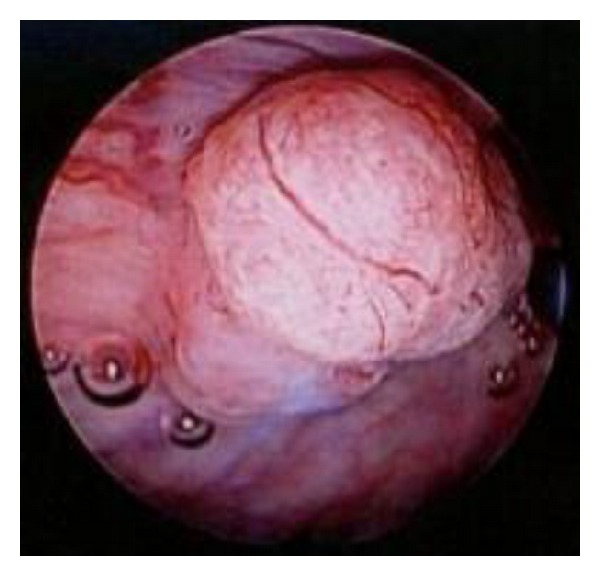
Nodular, broad-based tumors observed in the anterior wall of the urinary bladder.

**Figure 2:**
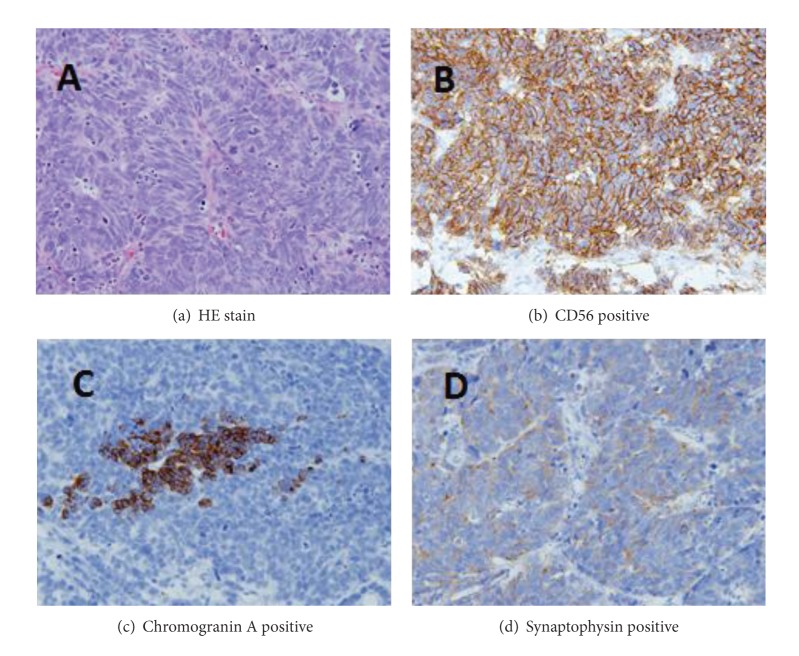
Histological examination.

The patient was a 84-year-old man. From 2003 to 2005, he underwent several TURBT (all of which were noninvasive and low-grade urothelial cancers: pTa). In May 2011, cystoscopy revealed a nodular, broad-based tumor on the anterior wall (Figure 1), and recurrent bladder cancer was diagnosed, for which he was admitted to our hospital for his surgery. TURBT was performed the day after hospitalization. Pathological diagnosis revealed cancer cells with a poor endoplasmic reticulum and elliptical nuclei rich in chromatin with medullary solid growth patterns. A rosette-like structure was also observed with an extremely high rate of mitosis.Immunohistochemical analysis demonstrated that tumor cells were positive chromogranin A, synaptophysin, and CD56 (Figure 2). Thus, he was diagnosed with LCNEC and urothelial carcinoma, G3, INF*γ*, pT1, ly0, and v0.

Since metastatic bladder cancer was also suspected, a computed tomography scan was performed to search for a primary lesion. A nodular shadow was detected in the upper lobe of the right lung, and on diagnosis of primary lung cancer, he underwent partial resection of the upper lobe in the department of respiratory surgery of this facility.

Tissue analysis revealed adenocarcinoma, so he was diagnosed with primary bladder cancer (LCNEC and urothelial carcinoma) and primary lung cancer (adenocarcinoma).

We thought to perform total cystectomy. However, after his surgery of lung cancer, his general condition became worse. Therefore, we have observed his condition strictly. After TURBT, he has had no sign of recurrence for 8 months.

## 3. Discussion

LCNEC is one group of neuroendocrine carcinomas in the lungs and bronchial tubes that was first reported by Travis et al. in 1991 [2]. Since then, it has been occasionally reported in various organs such as the uterus, thymus gland, stomach, bile duct, mammary gland, prostate gland, kidneys, and urinary bladder [3–7].

Histological characteristics of LCNEC include the following: (1) growth patterns such as organoid, trabecular, and rosette, (2) large polymorphic tumor cells, low N/C ratio, and crude chromatin with obvious nucleolus, (3) high rate of mitosis, (4) necrosis, and (5) neuroendocrine tumor characteristics present in immunohistology and electron microscopy.

Occurrence of LCNEC in the urinary bladder is extremely rare and, to the extent of my research, there are only 18 cases to date including this case. In a report compiled by Martin et al. [8], out of 17 cases, those with only LCNEC accounted for approximately half (11 cases), and those combined with other tissue types were the other half (9 cases). The most common other tissue type was urothelial cancer, followed by adenocarcinoma, squamous cell carcinoma, and sarcomatoid urothelial cancer. All subjects were diagnosed with macroscopic hematuria as the major complaint. Furthermore, they were mostly invasive cancers existing with distant metastasis at diagnosis. Clinical presentations resemble small cell carcinoma, and the incidence of recurrence or development is high, even if multimodal treatment is performed. While the effects of chemotherapy are unknown, Akamatsu et al. reported on a subject that underwent total cystectomy and etoposide, carboplatin combined chemotherapy for muscle-invaded LCNEC (pT2), and had no recurrence for 16 months [9]. They suggest that in the event of localized invasive cancer, early diagnosis and multimodal therapy may be a permanent cure. However, there is no standard chemotherapy for LCNEC, even for lung cancer as well. 

Factors behind the development of urinary bladder cancer with neuroendocrine function are unknown at present; however, there are a number of theories [10, 11]. One theory is that it originates in multipotent stem cells, and another is that it originates in submucous neuroendocrine cells, normal urothelium, or urinary tract epithelial metaplasia [12]. The former is sometimes supported in combination with other various tissue types. Abenoza et al. also hypothesize that neuroendocrine carcinoma of the urinary bladder originates in the urachal epithelium [13]. In this case, we suspect that development from pluripotent stem cells combined with urothelial cancer, as well as the underlying neuroendocrine cells beneath the mucous membrane, became cancerous and manifested.

This case was found by routine follow-up cystoscopy, so it could be diagnosed relatively early. However, there are many areas regarding LCNEC prognosis and treatment that remain unknown, and we believe that close follow-up monitoring is necessary.

We managed a case of LCNEC of the urinary bladder. This disorder is extremely rare, with many unclear points regarding treatment and prognosis. We believe this case to be valuable for the readers of this journal.
